# “We Made the Rule, We Have to Stick to It”: Towards Effective Management of Environmental Tobacco Smoke in Remote Australian Aboriginal Communities

**DOI:** 10.3390/ijerph10104944

**Published:** 2013-10-11

**Authors:** Jan Robertson, Boris Shane Pointing, Leah Stevenson, Alan R. Clough

**Affiliations:** 1 Tropical Medicine & Rehabilitation Sciences, School of Public Health, James Cook University, Cairns, Queensland 4870, Australia; E-Mails: leahstevenson@live.com.au (L.S.); alan.clough@jcu.edu.au (A.R.C.); 2 Midwifery & Nutrition, School of Nursing, James Cook University, Cairns, Queensland 4870, Australia; 3 Cairns Institute, James Cook University, Cairns, Queensland 4870, Australia; E-Mail: boris.pointing@jcu.edu.au

**Keywords:** aboriginal, Australian, remote communities, environmental tobacco smoke, smoke-free policies

## Abstract

Smoking prevalence in remote Australian Aboriginal communities remains extraordinarily high, with rates reported of up to 82%. Widespread exposure to environmental tobacco smoke (ETS) is exacerbated by overcrowded housing. Implementation of existing smoke-free policies is challenged by the normalization of smoking and a lack of appropriate regulation resources. This paper celebrates a grassroots approach to control of environmental tobacco smoke (ETS) in these settings. We report on selected findings from a tobacco intervention study in Arnhem Land, Northern Territory in 2007–2012. In community-level tobacco use surveys at baseline (n = 400 ≥ 16 years), participants reported concern about the constant exposure of non-smokers to tobacco smoke. Suggestions for action included restricting smoking in private and public spaces. We selected three case studies illustrating management of ETS from observational data during the study’s intervention phase. Using a critical realist approach, the context and mechanisms that contributed to specific strategies, or outcomes, were examined in order to develop a hypothesis regarding more effective management of ETS in these environments. Our results suggest that in discrete, disadvantaged communities, enhanced local ownership of smoke-free policies and development of implementation strategies at the grassroots level that acknowledge and incorporate cultural contexts can contribute to more effective management of ETS.

## 1. Introduction

The prevalence of smoking in the general Australian population is 15.1% [1]. Similar to Indigenous peoples of Canada, the United States of America and New Zealand [2], smoking rates of Australian Aboriginal and Torres Strait Islander peoples, while declining, are more than double the general population rate at 45.1% [1]. Additionally, smoking rates in remote Aboriginal communities have changed little over the past 20 years, remaining extraordinarily high, up to 82% [3]. In these communities domains of extreme disadvantage include especially poor health, education and low employment, all exacerbated by geographical isolation. Also overcrowded houses, coupled with very high smoking rates, point to health impacts of environmental tobacco smoke (ETS) which may be considerably worsened [4].

The current tobacco policy environment in Australia is unprecedented, featuring efforts to reduce the high rates of smoking among Australia’s Indigenous peoples. We previously reported that key stakeholders in the “Top End” of the Northern Territory (NT) considered smoke-free policies in particular, as a major opportunity to reduce tobacco use in these unique settings [5]. Spaces linked to these policies were identified as workplaces and public spaces and people’s homes. A key challenge to maximizing this opportunity is the lack of resources to ensure local compliance with policies that have been developed far away, at jurisdictional and regional levels.

This paper provides suggestions to encourage more smoke-free spaces in marginalized and disadvantaged communities, including effective implementation of smoke-free policies. The paper will use data collected during the Top End Tobacco Project (TETP), a recently completed five year tobacco intervention study in three remote Aboriginal communities in Arnhem Land, located in the Top End of the NT.

A significant component of this intervention study comprises a process evaluation to understand what works to reduce tobacco use among indigenous Australians in remote community settings. The TETP aims to understand not only what works but for whom and in what circumstances, consistent with a Realist Evaluation approach [6]. To frame this evaluation, in this paper we formulate the following hypothesis:

*In discrete disadvantaged populations*

*where there is high prevalence of tobacco use and tobacco use is normalized,*

*management of ETS will be more effective*

*if policies and implementation strategies are developed at the grassroots level*.

We also seek to align this hypothesis with existing policy frameworks. To do so, we use already published and further empirical data from the TETP, and the Critical Realism methodology to understand the mechanisms and contexts regarding the local development, implementation and regulation of strategies to manage environmental tobacco smoke in shared spaces in these communities.

## 2. Methods

### 2.1. Critical Realism

The Critical Realist tradition of social inquiry has a long history since Bhaskar first introduced the concept in “A Realist Theory of Science” in 1978 [6]. The aim of Critical Realism is to identify the causal mechanisms which produce patterns, the data regularities identified through qualitative and quantitative scientific methods [7]. In this paper we apply this Critical Realist method to the qualitative and quantitative findings of the TETP, and the policy and cultural environments to reflect on one central question: “What CONTEXTS and MECHANISMS contributed to the OUTCOMES of the identified ETS control strategies in each community?”

**Figure 1:**
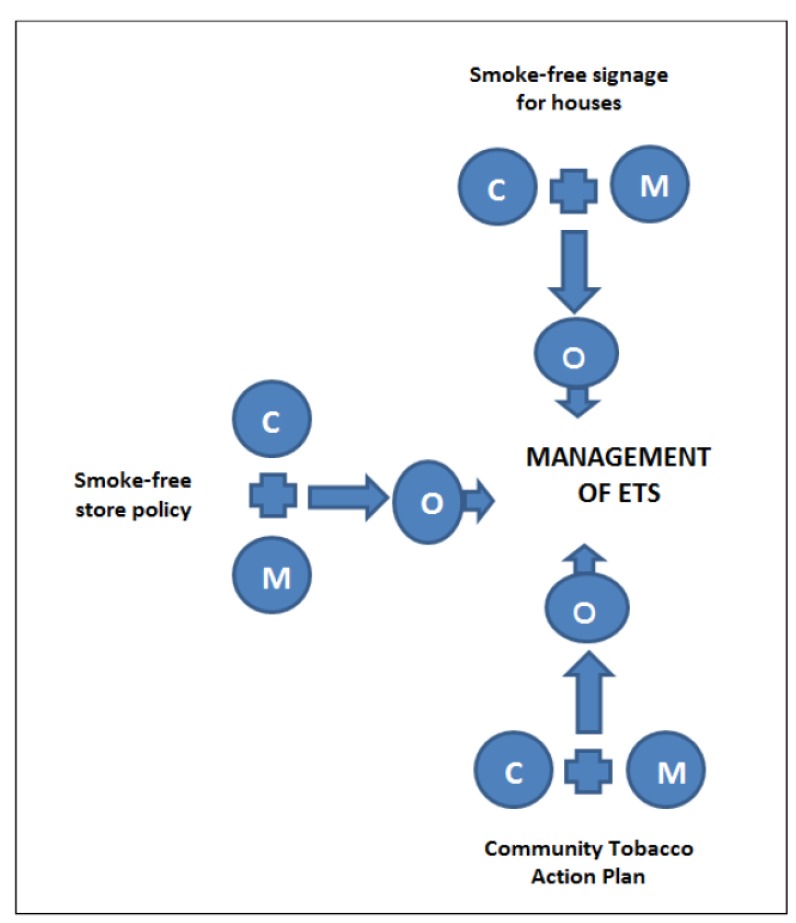
Logic diagram of case studies analysis (after Pointing 2012).

C = context; M = mechanisms; O =outcome

Using Koenig’s approach embedded in critical realism we use case studies to consider this hypothesis [8]. Three examples of management of environmental tobacco smoke were selected for case studies: (1) the development of smoke-free signage for homes in the three participating communities; (2) the development and implementation of a smoke-free policy for a remote community store area; and (3) development of a community Tobacco Action Plan with a community-determined focus on passive smoking. Exploring the case studies we seek to pull apart and understand the contexts and causal mechanisms in which they operate to explore the above hypothesis. The contexts explored in detail below include policy, geographical and cultural settings surrounding tobacco use and impacting on a particular outcome, that is, the management of ETS. Following the identification and deconstruction of the contexts and mechanisms, we apply Realist Evaluation [6]. A logic diagram [9] depicts this process showing the relationship between CONTEXT, MECHANISMS and OUTCOMES (Figure 1).

### 2.2. Settings

#### 2.2.1. Policy Environment

The general policy environment in which the TETP took place was tumultuous (Table 1). In response to the report released in 2007 on the sexual abuse of Aboriginal children in the NT, *Little children are sacred*, a national emergency response was announced [10]. The report recommended collaborative efforts between the Northern Territory and Australian governments and a commitment to consultation with Aboriginal communities to address this complex issue. Despite this commitment the Australian Government quickly imposed measures affecting approximately 45,500 Aboriginal people living within “prescribed areas” [11]. These included alcohol restrictions; banning possession of pornography, compulsory health checks for children (with logistical support supplied by the army), compulsory income management for those receiving welfare payments and control of townships through leasing of assets including housing. Related legislation included the highly controversial suspension of the Racial Discrimination Act. Despite some community support for some of the measures, the implementation of these with little consultation badly damaged the relationship Aboriginal people have with the governments of Australia and contributed further to a sense of disempowerment [11].

On the heels of this major policy change and the social upheaval that followed came reforms of the Local Government structure in the NT in 2008. Through the merging of local councils into shires, the number of Local Governing bodies were reduced from 61 to 15 [12]. Local councils, formerly with representation of each of the local traditional owner clans, have transitioned to representation by a few elected local representatives with governance located in regional centres. Many local clan leaders perceived this as the removal of decision-making beyond their sphere of influence [13].

**Table 1:** Timeline of key milestones of the Top End Tobacco Project aligned with selected national and jurisdictional policies impacting on remote Aboriginal communities.

Date	Top End Tobacco Project progress	Selected national and jurisdictional policy development and implementation
2007 Jun		Australian Government announces the NT Emergency Response (NTER) to protect Aboriginal children from sexual abuse. Includes deployment of army in communities
Jul	Community engagement visits commenced	
Aug		Legislation in support of NTER passed including *Welfare Payment Reform* establishing compulsory income management and suspension of the *Racial Discrimination Act 1997*
Nov		Change in federal government
2008 Feb		Prime Minister delivers Apology to Australia’s Indigenous peoples
Mar		Statement of Intent between the Government of Australia and the Aboriginal and Torres Strait Islander peoples to achieve equality in health status and life expectancy by 2030
Mar		Federal Government commits $14 million to address high smoking rates among Indigenous peoples
May	Community baseline surveys commenced	
Jul		NT Local Government reforms reduce 61 local governing bodies to 16
Aug	Community feedback of survey results commenced Tobacco Action Group formed in Community 1 Intervention components commenced	
Dec		National Partnership Agreement (NPA) on Closing the Gap in Indigenous Health Outcomes signed NPA on Remote Indigenous Housing signed
2009 Feb	Baseline surveys completed	
Mar		NT Tobacco Summit develops NT Tobacco Action Plan
Jul		NT Health Department implements Smoke Free Policy for all services & facilities
2010 Feb		National Coordinator to Tackle Indigenous Smoking appointed Start building new houses Community 1 Start building new houses Community 3
Mar	Smoke-free (SF) house signage Community 1	Formal signing of Local Implementation Plan Community 3
May		Announcement of anti-tobacco workforce targeting Indigenous peoples. 82 initial positions nationally
Aug	Community follow-up (FU) surveys commenced	
Nov		Formal signing of Local Implementation Plan Community 1
2011 Mar		Formal signing of Local Implementation Plan Community 2
Apr		Start building new houses Community 3
Jun	SF house signage Community 2 SF store opens Community 2	
Aug	SF signs Community 3	
Sept	FU surveys completed	
2012 Jun	Community 1 Local Reference Group (LRG) reviews Local Implementation Plan, decides to focus on passive smoking	
Aug	FU survey feedback completed Community 1 LRG & TETP workshop to develop Tobacco Action Plan (TAP). TAP endorsed at LRG General Meeting	

In a commitment made in 2008 to “Closing the Gap” in disadvantage between Indigenous and non-Indigenous Australians, the Council of Australia Governments set up National Partnership Agreements (NPA) between the Commonwealth of Australia and the states and territories [14]. Relevant to this paper is that the agreements include the National Partnership Agreement on Remote Service Delivery (NPARSD) and the National Partnership Agreement on Remote Indigenous Housing (NPARIH). The NPARSD encourages active participation of community members through the development of Local Implementation Plans (LIP’s) to improve coordinated service delivery and accelerate local development. Members of participating communities are represented by their Local Reference Group (LRG), which provide opportunity for more thorough representation of clan groups following the implementation of local government reforms. Support and guidance is provided to the LRG’s by the paid Community Engagement Coordinators (usually non-local and non-Indigenous) and the paid Indigenous Engagement Officers (usually a local Indigenous person). These positions report to Regional Operations Centre representing the interface of both commonwealth and jurisdictional governments [15]. The LIP’s focus on “Closing the Gap” domains of early childhood, schooling, health, healthy homes, economic participation, community safety and governance and leadership. A reduction in tobacco use has been identified as a priority area for action. The NPARIH, also relevant to this paper, has committed to building nearly 1,000 new houses, rebuilding and refurbishing almost 3,000 others in 73 remote communities and town camps across the NT by the end of 2013 [16].

Specific tobacco control policy development at both the commonwealth and state/territory level has been dynamic and unprecedented. In 2009, the Australian Government committed $100.6 million for the Tackling Indigenous Smoking program over the next four years. The program included funding for tackling smoking and healthy lifestyle teams across 53 regions. Further commitments totaling $37.84 million came from the states and territories [17]. The current NT Tobacco Action Plan (2010–2013) includes a strong focus on community-level interventions including programs to reduce passive smoking [18].

#### 2.2.2. The Top End Tobacco Project

The Top End Tobacco Project (TETP) was a multiple-component intervention study to reduce tobacco use in remote Aboriginal communities. Teams of 2–4 researchers travelled to each community for 4–5 days on a quarterly basis. Paid local co-workers facilitated local introductions and participant recruitment, enabling the team to manage local cultural protocols while also acting as interpreters. Special translation services were mostly provided by paid local linguists. Self-reported tobacco use in the population was measured in each community at baseline (total Indigenous population > 16 years = 2,319). Previously published data reported extremely high prevalence of current tobacco use among those aged 16 years and over (n = 400): Community 1: 71%; Community 2: 78%; and Community 3: 82%. As a component of the project’s translational research approach [19,20], we ensured the data collected during the research was made immediately accessible to community members and relevant stakeholders including local and regionally based service providers. Results of this survey [3,21,22] were comprehensively fed back to community members in a culturally-relevant and conceptually meaningful manner, as described elsewhere [13]. Information on the health impacts of tobacco was also included at the request of community advisors. We also presented comparisons between national and community level smoking rates which clearly challenged the normalization of such high prevalence. Three discussions arising from the face-to-face feedback of these results at the community level were able to inform intervention components which included: capacity building among local health staff to prevent tobacco uptake and provide cessation support; place-based approaches with a strong focus on workplaces; and mobilisation of community resolve to implement local policies and strategies to reduce tobacco use including information resources in local languages. To date, only preliminary analysis of the follow-up survey data has been undertaken. This reported little change in prevalence but captured substantial changes in participants’ attitudes to smoking. Our observational data demonstrates, through the selected case studies, that this included a greater interest in increasing smoke-free spaces in each community. In order to develop and examine the hypothesis we used the following data sources:

(1) Further selected findings from community surveys of self-reported tobacco use undertaken in 2008/09 in the three remote communities described above. Participants (n = 400 ≥ 16 years) were opportunistically recruited from community members, using quotas to reflect age and gender balances. Those current and former smokers who had made quit attempts were asked to provide information about relapse. They were also asked what their concerns were about tobacco use in the community and to provide suggestions for actions to address these concerns. Using structural coding, responses were grouped into categories agreed upon by authors JR and LS.
(2) Field notes of direct observations, including transcripts of community-level discussions relating to ETS, made during community visits by the research team over the five year period of the TETP. Three case studies relating to the management of ETS were chosen for this paper in order to try and represent activities across the study communities.

#### 2.2.3. Setting

**Figure 2:**
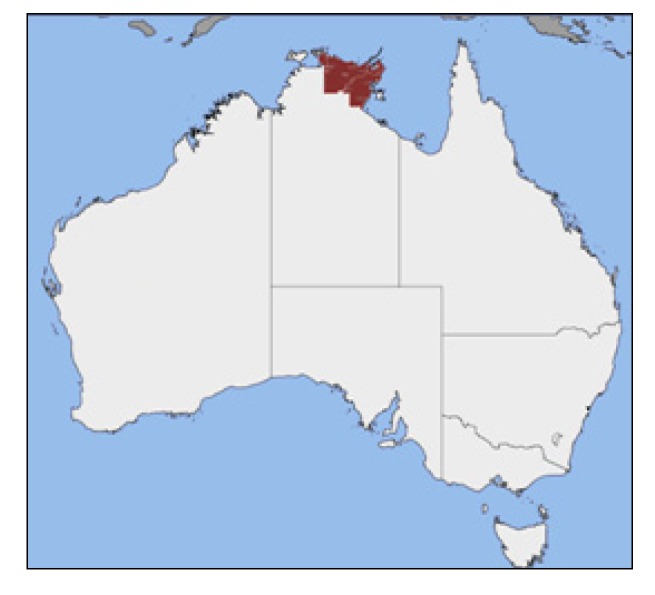
Location of Arnhem Land, Northern Territory, Australia (area shaded in red).

Arnhem Land is located in the north eastern corner of the “Top End” of the NT (Figure 2). The study communities have a combined population of 3,100, and are located in isolated corners of Arnhem Land, one of them on an island. Covering 97,000 km, the region is almost twice the size of England but with a population of approximately 25,000 [23]. Entry into the region to travel or work requires a permit from traditional owners of the land. In the region’s tropical monsoon climate, many remote communities can be isolated by flood waters for over six months of the year. Two of the communities are “dry”, that is, they do not have legal access to alcohol locally. The third is in a liquor restricted area but has a social club licensed to sell liquor on site within strict parameters governed by both community rules and the NT’s liquor licensing commission. Substance misuse has been an ongoing concern in Indigenous communities in the NT. Within the region of the TETP study communities, use of cannabis, alcohol, kava and sniffing petrol bring further health and socio-economic burden to these already disadvantaged populations [24]. Cannabis is widely used across the study region, usually smoked with a mix of tobacco, reinforcing continued tobacco use [25]. 

#### 2.2.4. Cultural Setting

In these isolated settings, many languages and cultural practices have been retained. Illness is often attributed to malevolent magic [26]. Management of shared space takes place within a complex framework of cultural law, including a kinship system which codifies communication within and between communities. Across the Top End of the NT, including the three study communities, a concept of balance underpins the conceptual division of much of the known world into moieties or halves: *dhuwa* and *yirritja* [27,28]. People are also *dhuwa* or *yirritja*, and further classified according to their position in the kinship system. At a general level, kinship is reckoned through a classificatory system which, in eastern Arnhem Land, is known as *mӓlk* [27,28]. The system of *mӓlk* initially distinguishes an individual’s position in the system, which is known by everyone, young and old. Space does not permit a full description of how *mӓlk* operates. However, relevant to this paper, once *mӓlk* is established, all relationships with others who also have *mӓlk* is predetermined and can be reckoned upon first encounter, including whether one is *dhuwa* or *yirritja*.

Rules around communication across segments within *mӓlk* are inherent to the system. These rules provide the protocols for respectful communication across all groups at all times. There are rules regarding avoidance of direct communication with, especially, one’s siblings and “poison cousin”, *i*.*e*., the mother of your wife. Requests from one’s brother or one’s uncle (from the perspective of a male) for resources, including tobacco or the money to buy it, are met with little or no questioning. This is within a context of demand sharing where everyone will share in a surfeit of a resource and all will share similarly in a dearth. Sharing is a powerful force as *gӓ ngarali* means in Djambarrpuyngu language, “give me tobacco”, as much a command as a request. Our team was told by community elders that *ngarali* is a word which recalls the startling experience of an initial nicotine rush in people unfamiliar with its use. *Ngarali* also describes the experience of eating the very rich meat of a particular sea turtle at a time of the year when it is regarded as especially rich in flavour and nourishment with a mild stimulant effect. For tobacco, in eastern Arnhem Land, *yirritja* people are the keepers of the knowledge regarding *ngarali*, as *ngarali* is also *yirritja*. Hence a *yirritja* man will refer to *ngarali* as his *waku* (son) [29]. “That’s my *ngarali*” is a statement of kinship, or personalised relationship people have with tobacco, as much as it is a statement of ownership.

The land and environment and all spaces within it are also *dhuwa* or *yirritja*. However, land and the places it contains is managed very carefully through a complex overlay of land ownership reflecting the interests of a local land-owning group [30]. Control of all aspects of the land is vigorously enforced and defended by the group. Access to land and the places and resources it contains is by respectful negotiation with the owner. Abiding by the conditions set by the landowner is paramount in this lorefull and tightly regulated system. Arnhem Land people are very much accustomed to operating within rigid frameworks of rules, lore or *rom*, which include the management of environment and land. As with many remote Indigenous community populations, inhabitants have two systems of law to manage: their cultural law and the law of the dominant culture, that is, Australian parliamentary and common lore.

## 3. Results

### 3.1. Baseline Survey Data

The proportions in the total Indigenous population (n = 2,300) aged 16–29 years (47% m, 43% f) and aged ≥30 years (53% m, 57% f) were similar to the proportions in the sample aged 16–29 years (39% m, 42% f) and ≥30 years (62% m, 58% f) (*p* = 0.064 for males, *p* = 0.428 for females, respectively). 

Italics are used to denote direct quotations from community members and other significant stakeholders.

#### 3.1.1. Quit Attempts and Relapse

Despite the high prevalence of tobacco use across the communities, well over half of the current smokers were considering or actively trying to quit [3,13]. Of the 183 current and former smokers who had made quit attempts, 60% (n = 109) provided information about relapse (data not shown). Reasons for relapse were coded into categories which were ranked according to frequency of mention. In descending order of frequency of mention the reasons cited for relapse were: cue exposure, alcohol use and peer pressure. *Cue exposure* was by far the most common reason for relapse, cited by 47% (n = 51). Examples of this included close proximity to other smokers: “*started (smoking) again seeing* *people smoking and inhaling fumes of smoke*”; “*too many people smoking around me*” and the ubiquitous custom of sharing smokes: “*because sharing and starting up others smokes for them, now I’m smoking full time*. *I* *try to stop but it*’*s hard*”. Many participants had been able to quit smoking when they left their community and visit or work in places where the prevalence was lower “*After going away for training I came back to my job (in the community)* *and started smoking again*”; “*I started when I came back from boarding school*” or there was limited access to tobacco “*I started smoking again when I came back to the community from the outstation*”.

#### 3.1.2. Community Concerns about Tobacco Use

Despite the high prevalence of tobacco use, over 300 survey participants, including smokers, expressed concerns about tobacco for either themselves or the community as a whole (Table 2). The impact of tobacco use on health was mentioned most frequently as an area of concern, particularly cancer and cardiac disease followed by worries about exacerbating asthma, and concerns about *short wind* (emphysema). Community members were also clearly dismayed about the decreasing age of uptake of tobacco use among children. The average age of uptake was 17 years (data not shown) with a range of 4–38 years. Passive smoking was the third most frequently mentioned area of concern. Smokers and non-smokers alike commented on the pervasiveness of tobacco smoke “*Everywhere is smoke*”. “*Sometimes there is too much smoke*”; “*I worry about breathing in other people’s smoke*”. Most commonly mentioned were worries regarding the exposure of babies, small children and “*old sick people*” to ETS: “*I don’t like smokers near my baby*. *The smokers know it’s poison*. *I make them stop smoking before they hold my baby*.” “*Young pregnant women smoking*, *newborn babies passive smoking*”. More females than males expressed concern about passive smoking.

#### 3.1.3. Community Suggestions for Action

Nearly 200 participants made suggestions as to what might work to reduce tobacco use in their community (Table 2). Five participants (four in Community 1) provided comments emphatically stating that dealing with smoking was a matter of individual choice about quitting, not offering suggestions about population approaches. “*People make their own choice to stop—none of our business what others do*”. For the purpose of this paper only the theme of smoke-free spaces will be discussed. This was ranked fifth in the top five themes identified and frequently linked with the pervasiveness of smoke: “*Everywhere is smoking—stop that*”. Suggestions for management of smoke-free spaces included private as well as public and workspaces “*There should be no smoking in front of the shop, ban smoking at clubs and pubs*. *Not in the house or car with kids*”; “*Staff need to smoke outside*”. Some people specifically mentioned linking the management of smoke-free spaces with encouragement for smokers to quit “*Encourage people, you better stop ngarali, as I do*. *Always ask people to smoke outside the house and not in front of kids and also in the car*”; “*More activities like workshops on quit smoking*. *Develop other smoke-free areas, space*s”. Women saw management of smoke-free spaces more frequently as an opportunity to address concerns about the high prevalence of tobacco use in the communities. This is in keeping with other data from the same survey reporting that women were more likely to restrict their smoking in selected environments such as houses, cars and the workplace (data yet to be published).

There was a greater proportion of people ≥ 30 years who provided responses to the survey questions above (sections 3.1.1: 62%, 3.1.2: 66%, 3.1.3: 70%) with similar proportions of males and females in this age group.

**Table 2:** Top End Tobacco Project community baseline survey: participants’ responses to questions regarding (i) their concerns about tobacco use and (ii) suggestions to reduce tobacco use.

Community Concerns about Tobacco Use/Smoking				
Occasions of Mention				
**Most frequently occurring themes**	**Female**	**Male**	**Total**	**Selected participants’ comments**
	(n = 136 */194)	(n = 166 */206)		
**Health (own or others)**	47	58	105	*Shortwind when running*. *Worry about dropping dead*.
				*Dangerous—lots of people short of breathing*. *Asthma*. *Cancer*. *Pregnant smokers have skinny babies*
**Young age of uptake/kids smoking**	33	29	62	*Kids starting to smoke younger than 10*
				*Little kids smoking…watching and copying others around*
**Passive smoking**	18	10	28	*I worry about breathing in other people’s smoke*
				*Young mums smoking around babies*
**Poor role model**	6	6	12	*Smoking next to kids, that’s not good and they look at us smoking*.
				*Little kids…watching and copying others around*
**Addiction**	3	8	11	*We can’t stop bakki (tobacco), never*
				*I can’t sit without ngarali (tobacco)*
**Community Suggestions to Reduce Smoking Tobacco Use/Smoking**				
**Occasions of Mentions**				
	**Female**	**Male**	**Total**	
	(n = 113 */194)	(n = 138 */206)		
**Health promotion**	25	49	74	*Never get kids to light up (others cigarettes for them)*
**Activities**				*Need to have pictures to see that ngarali (tobacco) is a killer.*
				*Make resources in Yolngu Matha (language)*
**Quit support**	19	26	41	*Need to learn about ways to quit*
				*Provide gums and patches. We have enough information now. There is no need for more. Just need to give up*
**Supply reduction**	19	13	32	*Tell factory not to sell cigarette because it kills people’s hearts and minds*
				*Should ban tobacco in the community*
				*Ban smokes from the shop*
**Diversionary activities**	19	13	20	*Go out bush long term. To an outstation for 3 or 4 weeks and give up there. Lots of hunting and fishing and collecting bush tucker. Teach the kids to make spear then get kids off cigarettes*
**Smoke-free spaces**	12	3	15	*First step is to encourage people to smoke outside—special smoking areas so the butts aren’t everywhere*
				*Make the houses and streets smoke-free*

* Number of baseline survey participants of total (by gender) who provided a response.

### 3.2. Observational Data from Intervention and Follow-up Survey Phase

Feedback of the baseline survey results and preliminary results of the follow-up survey was a catalyst for discussions on strategies to reduce the impacts of tobacco use. In Community 2 a conversation about the health impacts of smoking took place with women who, respectively, were the local Housing Officer and the Environmental Health Officer (EHO). They decided: “*instead of you coming here, we can do these things*. *Carry stories about passive smoking to houses, encouraging smoke-free homes and making smoke-free signs*”. The EHO, who also provided education about nutrition and tropical infectious diseases, stated “*the tobacco message will be the most important message to bring to homes*”. They discussed incentives that the local councils could provide for community members to declare their houses smoke-free, such as garden implements, heaters for the winter or “power cards” (vouchers for payment of electricity usage). In Community 3 community members discussed policies regarding the management of smoke-free spaces outside of their communities “*People are thinking a bit more*. *There are more government rules*. *Even when we go to the pubs (outside the community), people ask*: *where is the smoking zone, where can we smoke?*” One person, recently returned from a nearby town, wondered why there were not more similar stringent rules about smoking in his community.

We reflected on baseline survey results reporting on the most commonly mentioned reason for relapse, that is, cue exposure, and community concerns expressed about exposure to ETS. Also that in spite of being seen as a major opportunity to reduce tobacco use and related harms, effective implementation of smoke-free policies in these communities was thought of as hampered by lack of strategies encouraging compliance [5]. Because we had, however, observed some community actions to manage ETS emerging throughout the life of the project, we wanted to gain an understanding of what lead to these actions. Since there is little reported about regulatory tobacco control efforts in these settings, our data lead us to developing our hypothesis:

*In discrete disadvantaged populations*

*where there is high prevalence of tobacco use and tobacco use is normalized,*

*management of ETS will be more effective*

*if policies and implementation strategies are developed at the grassroots level*.

We found three case studies within the project relating to management of ETS (see Case Studies 1–3) to explore this hypothesis. The case studies selected were those where there was a degree of community ownership and control over the activities and which resulted in tangible outcomes. The studies were chosen to ensure that an activity relating to management of ETS from each community was included.

**Figure 3:**
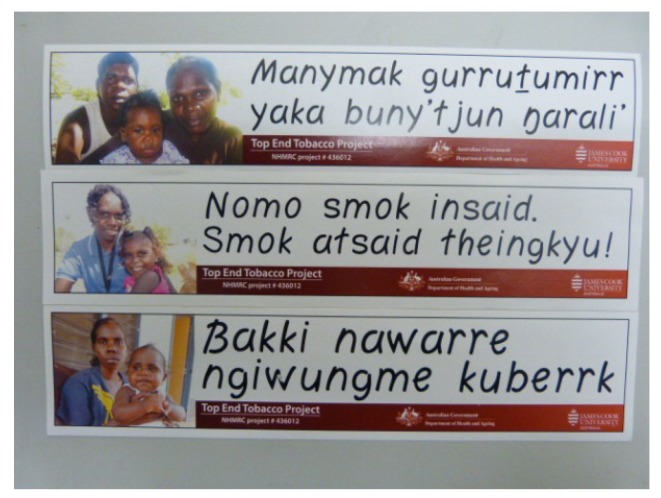
Local language stickers encouraging—smoke-free homes.

**Figure 4:**
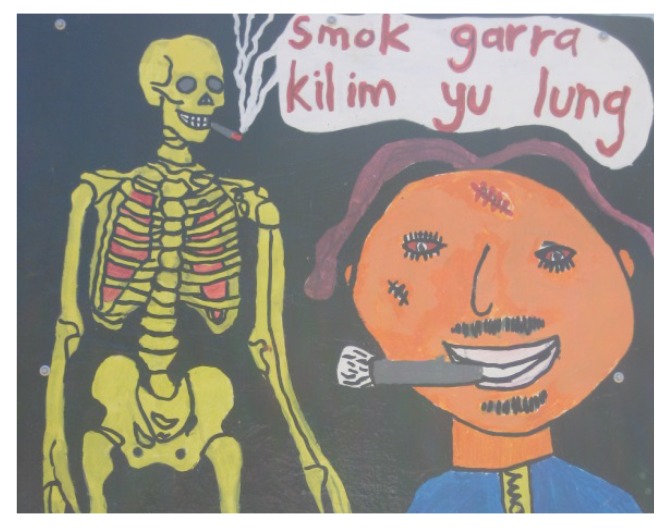
Mural at remote community school.

*Case Study 1*: *development of smoke-free signage for homes across the three remote Aboriginal communities (Communities 1, 2 & 3)* *participating in the Top End Tobacco Project* **(see Figure 3 and Figure 4)**

Our baseline surveys reported that the majority of smokers wanted to quit but as one participant noted: “*There isn’t much help here in the community for people wanting to quit*”. We wanted to understand the challenges faced by those smokers trying to quit in environments where they are “*surrounded by smokers*”. We provided intense quit support to self-selected smokers for up to ten days. After initial assessment and development of individual care plans we followed up participants in their homes and workplaces every second or third day. Early in 2010 in Community 1 (smoking prevalence: 71%) we visited a couple at home who stated they were managing to abstain during the day. They both worked in the local child-care centre, where smoke-free policies are universally respected in the communities. But coming back to their home, inhabited by several generations of family, many of who were smokers, they felt very tempted to smoke in the late afternoon and evening. Acknowledging the influence of the kinship system that ruled family relationships and communitions, we discussed the possibility of negotiating with others in the house about smoking outside the house and away from the verandah. They concluded that: “*The family will follow our rules*”. They also thought some signage in the house might help. Sitting together in the cool shade of an old tamarind tree we worked on the wording in Djambarrpuyngu language, one of four major Yolngu languages across the north eastern region of Arnhem Land. Due to enduring cultural relationships with tobacco in this community there is some reluctance for individuals to tell others to stop smoking. The resultant message was courteous rather than explicitly directive:

*Manymak gurrutumirr yaka buny’tjun ngarali* (Good/healthy family—no smoking tobacco).

Later in 2010, in Community 2 (smoking prevalence 78%), we showed a group of women the sticker during an informal discussion about encouraging people to smoke outside the home. They knew the family pictured on the sticker, and wanted something similar for their homes in Kriol, the lingua franca of their community which has several languages. Lively discussion about the message included comments: “*Don’t reject the person*, *reject the smoke*”. “*If you be nice no-one will listen*”. “*Can you talk to the housing mob and get them to build us smoking areas outside the house*?” The ladies came up with a no-nonsense message: 

*Nomo smok insaid*. *Smok atside theingkyu!* (No more smoking inside. Smoke outside thank you!).

Another group of women in this community also decided “*We can make a sign for the new houses, give them to all the heavy smokers to put up*”. “*We want all our family who smoke to smoke outside*. *We got kids inside, have a place in the yard for the smokers*”. Young women remembered anti-tobacco murals painted as part of a school project and thought the community should use those.

In 2011 in Community 3 (smoking prevalence 82%) there were nearly 90 new houses under construction in the community. One man told us: “*I don’t really want to quit*. *Next time you come back I will have my own house*. *I will be able to control my own space*. *I’ll be able to have a “no smoking” house and yard*”. Another, who told us he was “*desperate to quit*” stated: “*I cut down a little*. *I want to stop but it’s hard at home*. *My mother chews (tobacco) and my father smokes*. *They both spit*. *I want you to go and talk to them about not smoking inside*. *When I get into my new house I won’t have smoking inside*. *I might try to stop then*”. In a meeting with men in their workplace, we commented on the number of people wanting to make their new houses smoke-free for the sakes of non-smokers, especially old people, babies and small children. They agreed this was a good idea, and that there should be a smoke-free sign in their language, Kunwinkju, suggesting: 

*Bakki nowarre*. *Ngiwungme kuberrk* (Tobacco is no good. Smoke outside).

All the messages were checked by local linguists. We observed that the accompanying photos of local family members frequently elicited expressions of prideful recocgnition. In response to requests by both community members and service providers, and with funding from the Commonwealth Government, over 6,000 stickers were printed and distributed across Arnhem Land in regions appropriate to the languages.

**Figure 5:**
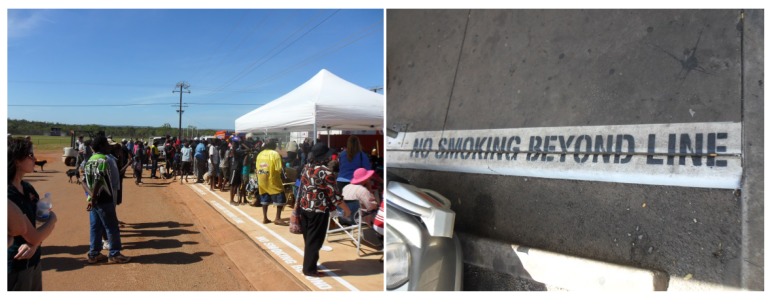
Opening of new smoke-free store precinct.

*Case Study 2*: *development and implementation of a smoke-free policy for store in a remote Aboriginal community (Community 2) participating in the Top End Tobacco project* **(see Figure 5)**

In the torrid tropical climate of the three study communities, the large verandahs of local stores afford protection from sun and rain. Popular gathering spaces, they are alive with the hustle and bustle of shoppers, their families, and those assembling to share a yarn, and often, a smoke. Although Territory-wide legislation bans smoking within two metres of the shop entrances, busy store managers said they had insufficient resources at that time to ensure these regulatory requirements were met.

In early 2010, while providing cessation support to a community member “desperate to quit”, the research team accompanied her to the store around mid-morning. The woman had managed to abstain from smoking since the evening before with the aid of nicotine replacement therapy. However, the smokers were about three deep along the verandah and she had shared a couple of “short ones” (shared cigarettes) before entering the shop. We realised that the almost daily visit to the local store would be overwhelming for many trying to quit. We reflected that clan leaders here had responded to feedback about the local smoking prevalence, particularly in comparison with the rest of Australia, with: “*We have to do something to narrow that gap*. *Even us leaders who smoke, we’ve got to show leadership too*”. We promptly shared this story with a local clan leader, who advised us to write to both the clan leaders group and the local store committee. We did this, citing some of the ideas that came up when we were talking to people around the community: “*With the new store owned by the Traditional Owners (TO’s) to be built in the community, maybe there could be a new smoking policy, for example, no smoking within 10 metres of the shopping complex*”; “*Smoking at the shop should be away from the verandah—the shop committee would make decisions like that*”; “*Go the political way with community by-laws, making new smoking rules about No Smoking areas*”. We also contacted the store management. Owned by the Australian government with an independent board of directors, the company returns profits from the store back to the community and is committed to the provision of healthy, affordable food to remote Indigenous communities. The company (head office) was already working to comply with tobacco legislation and also support healthy lifestyle choices for staff and customers.

In 2011 the new store and precinct opened, with a line designating the whole area smoke-free “*which has been well-received by local residents*”. At the time of our next community visit, the policy was actively being enforced by the store security officer, an elderly traditional land owner who sat outside the entrance beneath a No Smoking sign. When asked to provide some detail about how he ensured the policy was kept he replied: “*I tell people that we have a rule (smoke-free store)* that *we made*. *We made the rule*, *we have to stick to it… You need some-one to point the rule out*”.

*Case Study 3*: *development of a Community Tobacco Action Plan (Community 1) by a remote Aboriginal community participating in the Top End Tobacco Project*

The baseline community tobacco use survey and data analysis was completed in this community in 2008. We invited key community members and service providers to a presentation on a review of the project findings and asked them to advise us on (1) how to get these stories back to the community; (2) what actions might arise and (3) who should be involved in implementing these actions. The group agreed that the survey results were useful for all of the community and needed to be delivered in a way that people could understand. They decided that our team should feed the information back to the community “the same you collected it: work clan by clan, family by family, with co-workers from the community”. Suggestions for action included: “*Look for rupiah (money) to employ Yolngu to do house to house education, every house, clan by clan*, *using pictures and models*. *This needs to keep going to see change in smoking*”; “*(quit) support groups should be formed—away from the clinic because they are busy with other djama (work)*”. “*We need to make a Tobacco Action Group (TAG) for the community and homelands*” (small communities established to maintain connection with traditional, ancestral land). The majority of those attending agreed to be part of the TAG. However the group dwindled and folded within 6 months due to the lack of a local driver. 

In 2010 Tackling Indigenous Smoking funding was received by a regionally-based health service for two local dedicated Tobacco Worker positions in this community. We were able to assist with recruitment and capacity building of the male and female Yolgnu workers, being mindful of recommendations made for these positions in 2008 during the initial survey feedback discussions. The Tobacco Workers, in turn, collaborated with us on the TETP particularly during the follow-up survey and community feedback phase. However there was still need for a locally—driven, whole of community approach. In 2011 Community 1 signed off their Local Implementation Plan (LIP) for the National Partnership Agreement on Remote Service Delivery, including strategies to reduce smoking as part of a priority health area. On our final visit to this community in 2012, elements of the LIP were under review by members of the Local Reference Group who included the two community Tobacco Workers, the Community Engagement Coordinator (a former smoker) and the Community Engagement Officer (a community member and senior member of local government experienced in policy development). We were invited to attend a workshop to develop a Tobacco Action Plan informed by results of research undertaken by the TETP over the past five years. The desired outcome for the workshop was a plan that reflected the community’s desire to focus on strategies that addressed the impacts of passive smoking, particularly on children.

The workshop resulted in identification of the following key objectives: smoke-free homes and cars; provision of incentives for smoke-free homes; development of Designated Smoking Areas in public spaces; provision of tobacco information and education; review of smoke-free policies of all local organisations/service providers and alignment of community tobacco rules with jurisdictional tobacco legislation. Actions (strategies), required resources and key local and non-local stakeholders were identified. A participant noted: “*Today, this planning is closing the gap*. *Getting people together and trying*.” A local Health Committee was nominated to progress the actions identified in the Tobacco Plan. This plan was endorsed the next day at the general Local Reference Group meeting.

Using a critical realist approach we examined the contexts and mechanisms that produced outcomes relating to management of ETS (see Table 3).

**Table 3:** Analysis of three case studies using a critical realist approach to explore their context, mechanisms and outcomes.

Analysis of Case Studies Using Critical Realist Approach			
Study	Context	Mechanisms	Outcomes
**Case Study1**	Community 1 Couple wanting manage ETS in their home to support smoking cessation Ceremonial and cultural connections to tobacco Overcrowded housing Community 2 & 3: Wanting to manage ETS in their home due to concerns about passive smoking Wanting to take stronger steps to managing ETS as shifting into newly built homes	Quit attempt encouraged by smoke-free workplace policy Use of local language and images of community member Use of style of speech appropriate to the community Managing relapse prevention by reducing cue exposure Negotiation of physical boundaries to manage ETS in homes Funding supplied by commonwealth tobacco-specific taskforce	Smoke-free signage for homes Theme of signage replicated in other communities
**Case Study 2**	Community 2 Many community members visit store daily or more Store verandah viewed as public space for socialising Few public spaces shielded from the sun and rain Store managers reluctant to enforce legislation under NT Tobacco Control Act	Strong community leadership (translation to action) Local committees/groups with authority for tobacco action (Clan leaders group, Store committee) Collaborative approach (Clan leaders group, store committee, store management, research team) Store management actively committed to tobacco control Effective enforcement strategy included signage and a designated person	Smokefree policy for community store
**Case Study 3**	Community 1 Local Implementation Plans that require action on reducing tobacco use up for review. LRG (community representatives) decides to focus on “passive smoking”	Local Tobacco Worker LRG member—keeps tobacco on the group’s agenda Workshop developing tobacco action plan informed by recent tobacco research in community Clear sense of ownership of the TETP data by the community	Community Tobacco Action Plan targeting ETS

We examine the elements identified in the mechanisms to understand what has contributed to the specific outcomes (Table 3). Common across the case studies was the direct involvement of community members in development and/or and implementation of specific strategies. In Case Study One devices such as local language and images of local community members were used to increase salience of the messages. Particularly in Case Study Two we note a strong collaborative approach among stakeholders at the local level. Case Studies Two and Three involved the presence of local drivers (individuals or groups) who have either a mandate or interest to be actively involved in tobacco control efforts. Financial and human resources were available to support the above efforts. These included: the provision of funding to print and distribute the household signage; existing local committees to develop and ratify the store policy and a supportive store management that extended the position description of their security officer to include managing compliance with the new “no-smoking” policy that suited the cultural context. In Case Study Three national policy implementation related to closing the gap in Indigenous health supported tobacco control efforts through the provision of human resources. These were community-based Tobacco Workers and staff to support community representatives develop local implementation plans to improve remote service delivery. These plans specifically identified reducing tobacco use and related harms as a health priority for services. Local tobacco use prevalence data and information relating to tobacco use harms disseminated by the research team was utilized to inform community plans.

## 4. Discussion

At the time of the commencement of the project, the foremost context common to each community was the normalisation of an extremely high prevalence of tobacco use. Social, cultural and historical factors have embedded tobacco use in these settings, creating significant barriers to change. In response to community concerns, local and regionally based service providers with the core business of substance misuse have had their time consumed with addressing the more overt and immediate results of alcohol abuse and petrol sniffing. The lengthy lag-time of the onset of symptoms and signs of tobacco-related disease has made this substance less of a priority in these often crisis riven settings with limited resources forcing more focus on reactive responses rather than preventive strategies. However the baseline survey demonstrated that there was considerable awareness of tobacco related harms, particularly the respiratory and circulatory system effects, indicating some success for prior local health information initiatives. The baseline surveys also confirmed what had been found in similar settings over ten years ago, that among the majority of smokers there was a readiness to quit [31]. However, our surveys also reported there was still a paucity of knowledge among tobacco users about cessation techniques and limited access to local quit support [22], including the full range of cessation medications available in less remote environments [32]. 

Reducing tobacco use among the Indigenous peoples of Australia has been designated as a priority action area to reduce the gap in Indigenous disadvantage [33]. In an effort to similarly reduce health inequalities in England, smoking cessation services were set up targeting disadvantaged areas. An assessment of this strategy noted a “modest” impact, resulting in recommendations for more innovative cessation interventions and a call for a wider range of strategies relating to tobacco control [34]. The need for culturally appropriate cessation services has been identified for Indigenous populations [2,35] but these are extremely limited in remote community settings in Australia due to health services stretched by geographic isolation and lack of local capacity. As part of the TETP our team held workshops targeting local Indigenous Health Workers (IHW’s) throughout the life of the project, utilising reciprocal learning approaches to build health promotion and clinical capacity. We found, though, that management structures generally constrained both clinic and community-based IHW’s to brief interventions, rather than venturing into the area of intense quit support.

Recommendations to reduce inequalities in tobacco use in these and other disadvantaged populations include a comprehensive range of strategies including population-level approaches [36,37] rather than relying on clinical interventions with individuals [38]. These include: community interventions targeting youth; media campaigns, reinforcement of tobacco retail legislation, education about second-hand smoke particularly in homes and restriction of smoking in public spaces [37,39].

In other populations smoke-free policies in workplaces and public areas have been shown to reduce tobacco use [40,41]. In a study across four “affluent, western” countries, smoke-free public spaces were thought to be a catalyst for the adoption of similar policies for private homes, which in turn, was associated with increased quit attempts [42]. Although there is little published regarding similar courses of action and outcomes in discrete remote Aboriginal communities, Case Study 1 indicates that these international findings may also have some application in Australia. Access to new and less crowded housing has provided opportunity for greater control over private space. For some community members this has had a spillover effect of prompting new rules and behaviours around tobacco use, including smoke-free houses which may reduce consumption.

“*A policy which is embraced by a Minister, approved by Cabinet, announced publicly, but inadequately delivered is worse than no policy at all*…”.

Secretary of the Department of the Prime Minister and Cabinet [43]

It is one thing to have a policy and another to effectively implement it. Place-based smoke-free policy development guidelines, including smoke-free *maraes* [44] (New Zealand Maori sacred community meeting places) and Indigenous Australian workplaces [45], discuss the importance of evaluating the effectiveness of the policy and compliance. A vital component of effective enforcement of policies is the provision of public education in order to rally grassroots support for the laws, resulting in improved compliance [46]. In the study communities, the ongoing activities of the TETP substantially helped to increase community awareness of the impacts of tobacco use including passive smoking and may be considered as a sustained “*de facto*” public education campaign albeit limited to providing an understanding as to why such policies exist, Compliance strategies ideally should identify who is responsible for policy enforcement and also the process for dealing with any breaches [47]. We observed NT tobacco policy was generally poorly enforced in the participating communities, with little evidence of who was responsible for enforcement. Directed by regionally based supervisors, local managers shrank from what they perceived as a potentially contentious task [5]. But the case studies demonstrate that small, community-driven steps are being taken to limit exposure to ETS.

### Limitations

We could not use the follow-up data because it has not yet been fully analysed. Also, this is largely a descriptive paper (with no comparison group) therefore we are cautious with our recommendations. A possible further limitation was “fly-in, fly-out” approach by the research team, however over the life of the project the team was in the communities as consistently as many other visiting services. The five year time frame enabled the research team to build relationships of trust with community members and service providers, demonstrate preparedness to act on community recommendations, and have a sufficiently sustained presence to make the most of opportunities that presented themselves.

## 5. Conclusions and Recommendations

We conclude from the findings that management of ETS, including smoke-free policies, in discrete disadvantaged populations with high prevalence and normalization of tobacco use maybe more effective if implementation strategies can be considered at the grassroots level. Importantly, this can allow for the acknowledgement and incorporation of cultural contexts where appropriate. As described earlier in this paper, several aspects of the policy context common to the three participating communities have contributed either directly or indirectly to supporting efforts to reduce tobacco use in these settings. Reducing tobacco use had been viewed in communities as the domain of health services, but the NPA’s in particular provide an exemplar of the whole of government approach including collaboration with non-government organisations called for in Australia’s National Tobacco Strategy [17]. For the TETP this was a beautiful alignment of policy opportunities. At the grassroots level we have seen these policies provide opportunities to reduce tobacco smoking and its associated harms through collaborative efforts between local government, workplaces, educators, clan leaders and other community members. We have found these remote, discrete communities to be environments where new knowledge is shared quickly, leading to rapid adoption of novel practices. Successful practices are often quickly copied by other communities.

Based on the findings of this paper we make practical recommendations for community-level approaches to management of smoke-free spaces in these remote settings. Some of these may find application in other discrete and marginalised communities:

**Policies developed at a non-local (regional or jurisdictional) level:** get informed from the local level about potential barriers to policy implementation. Provide local people the opportunity to put some ownership on the policies through developing implementation strategies.

**Smoke-free workplace policies:** is there one? What do staff know about the policy? Review the policy in a workshop with local staff. Should the site be totally smoke-free? Or could this be better achieved in stages, that is, commence with designated smoking areas. Develop detailed policy implementation strategies including realistic time-frames, locally developed signage, information dissemination about the policy, its timeframes and enforcement. 

**Smoke-free homes:** are there Environmental Health Officers in the community? Ensure they are equipped to provide information about the harms of passive smoking (a hand-held expired breath carbon monoxide monitor is a great tool for engaging people in brief interventions [22]). For those living in public housing, Local Government and Housing organisations may be able to provide incentives for declaring their home smoke-free.

**Smoke-free public spaces:** in environments where there is traditional ownership of land ensure there is clarity of ownership and the landowner is involved in the decision making. This may impact on the location of designated smoking areas for example: where construction of a shelter against the elements is required.

**Local coalitions:** try to get tobacco onto the agenda of existing coalitions or forums of local key stakeholders. In smaller communities those with greater capacity are often the busiest and most burdened and may not be able to attend yet another meeting.

**Cultural issues:** ensure policies and implementation strategies acknowledge and incorporate local cultural conventions.

When “rules” about ETS management are made at the local level, these efforts need to be vigorously encouraged, appropriately informed and adequately resourced with consideration to staffing, skills and finances. More comprehensive reporting systems which are sensitive to community narratives about tobacco action may also do justice to capturing activities which, in our experience, are bound to include some very innovative practices that are worthy of sharing widely.
